# Experimental and *ab initio* studies of Co-doped ZnO nanophotocatalyst thin films for dye mineralization

**DOI:** 10.1039/d3ra04491b

**Published:** 2023-12-01

**Authors:** Ansa Latif, Muhammad Mohsin, Ijaz Ahmad Bhatti, Asif Ali Tahir, Muhammad Tahir Hussain, Javed Iqbal

**Affiliations:** a Department of Chemistry, University of Agriculture Faisalabad Pakistan ijazchem@yahoo.com m.mohsin618@gmail.com; b Solar Energy Research Group, Environment and Sustainability Institute, Department of Engineering, Faculty of Environment, Science and Economy, University of Exeter Penryn Campus Cornwall TR10 9FE UK; c Department of Applied Sciences, National Textile University Faisalabad 37610 Pakistan

## Abstract

Pristine ZnO and Co-doped ZnO photocatalyst thin films were fabricated on a ceramic substrate by spray pyrolysis. The optical, morphological and structural properties of the fabricated nanophotocatalyst thin films were analyzed using X-ray diffraction (XRD), scanning electron microscopy (SEM), energy-dispersive X-ray (EDX) spectroscopy and Fourier transform infrared (FTIR) spectroscopy. Operational parameters, including dye concentration, oxidant concentration, irradiation time and pH for dye degradation, were optimized by response surface methodology (RSM). The maximum degradation obtained was 93% under ideal conditions, such as pH 7, 3 h of direct sunlight irradiation, 30 mM concentration of oxidant and 10 ppm concentration of dye (MB). The evaluation of the extent of degradation was done using the UV/visible spectrophotometry technique. The reusability of the fabricated thin film was examined under optimized conditions. Density functional theory (DFT) with the B3LYP/LanL2DZ method was used for the theoretical modelling of the fabricated nanomaterials. The optimized structure, theoretical band gaps, IR spectra and Raman spectra of the fabricated pristine ZnO and Co:ZnO nanophotocatalysts were determined.

## Abstract 2

Pristine ZnO and Co-doped ZnO photocatalyst thin films were fabricated on a ceramic substrate by spray pyrolysis.

## Graphical abstract

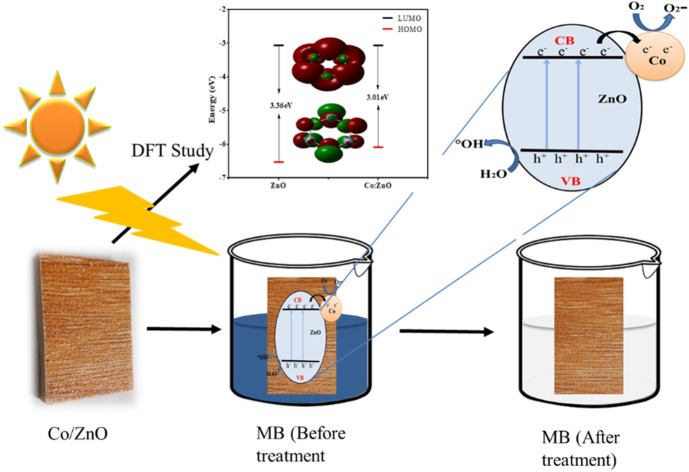

## 1. Introduction

Organic dyes released from textile, cosmetic, leather and paper industries have become a serious cause of poisoning of water resources.^1–3^ These dyes are difficult to decompose due to their complex structure. Dyes are extremely poisonous and capable of inducing various health problems.^3–7^

The harmful effect of dyes can be reduced by degrading them. Conventional methods, biological approaches, sonocatalyst processes and coagulation are not effective in degrading many organic dyes due to their highly stable chemical nature^8^ and less biodegradability. To solve this problem, modern-day techniques have been used recently, among which advanced oxidation processes (AOPs) have been found to be one of the best methods.^9–11^ Photocatalytic degradation methods are highly effective in degrading organic dyes that are resistant to biological degradation among AOPs.^8,12^

Photocatalysis is a potential approach for removing effluent dye molecules since it is affordable and works effectively under sunlight.^13–15^ Hydroxyl radicals are responsible for the degradation of dyes, and hydroxyl radicals are mainly generated in the presence of oxidants.^16^ Zinc oxide (ZnO) is the most preferred photocatalyst among semiconductor photocatalysts due to its significant photocatalytic activity, non-toxicity, wide band gap and cost-effectiveness.^17–19^ The quick recombination of charge carriers and the low efficiency in the visible range due to its wider band gap are two significant drawbacks of ZnO.^20^

As we know, the solar spectrum contains only 5–7% UV light but^21^ 46% visible light, and hence, it is convincing to bring the ZnO photocatalyst in the range of visible radiation to get better results. Various ZnO-based doped nanomaterials have been produced by combining with different transition metals and semiconductors to overcome these disadvantages, thereby boosting the photocatalytic efficiency of ZnO in the visible range.^22^ Doped ZnO nanoparticles act as effective trappers, improving the photocatalytic efficiency by enhancing the dissociation efficiency of photogenerated charge carriers and preventing their recombination.^23,24^ The doping of ZnO with transition metal ions successfully extends its photosensitivity from UV to the visible region, as it lowers the bandgap of ZnO. Selvaraj *et al.*^25^ showed that doping improved the degradation from 40% for pure ZnO to 93% for Gd-doped ZnO. L. Roza *et al.*^26^ demonstrated that bandgap narrowing from 3.28 to 3.26 by cobalt doping improved dye degradation from 67% to 80%.

Because of its comparable ionic radius, divalent state, various electronic states and highly soluble nature, cobalt is a potential dopant metal for ZnO among transition metals.^27^ Cobalt dopants are employed to create new energy levels in the bandgap that can be stimulated by visible light.^28^ In addition, cobalt is a remarkable partner of the ZnO nanophotocatalyst to enhance the photocatalytic activity by substituting the ZnO structure.^29^

Compared to various methods of synthesis of nanoparticles, spray pyrolysis is the best method for thin film formation because it is the most efficient and offers lower setup complexity with minimized waste production.^30^ ZnO and Co/ZnO thin films are produced by a spray pyrolysis process and their photocatalytic degradation activity against dyes has been investigated.^17,31^ A theoretical calculation of different compounds attracts much attention at present. Theoretical calculations have matched the experimental observations of metal oxides.^32,33^ Density functional theory (DFT) is one of the verified analysis methods, using which the properties of materials can be tailored efficiently.

In the current work, pristine ZnO and Co:ZnO thin films were fabricated on a ceramic substrate by spray pyrolysis. The synthesized compounds were characterized by XRD analysis, SEM, EDX analysis and FTIR spectroscopy to study their properties. The photocatalytic efficiency of Co-doped ZnO was investigated for methylene blue under sunlight and validated by the DFT-based theoretical results. Operational parameters including oxidant concentration, dye concentration, irradiation time and pH for dye degradation were optimized with response surface methodology (RSM). The evaluation of the degradation rate was done by the UV/visible spectrophotometry technique. The reusability of the fabricated material was checked under optimum conditions. Water quality parameters as COD, BOD and TOC were used to analyze the photocatalytic mineralization of MB.

## 2. Experimental details

### 2.1. Materials

The chemicals employed were all of analytical grade and did not require additional purification. Zinc sulfate heptahydrate (99.98%) and hydrochloric acid (98%) were purchased from Merck. Sodium hydroxide (98%), cobalt sulfate heptahydrate (99.98%), and hydrogen peroxide (98%) were purchased from Sigma-Aldrich.

### 2.2. Fabrication of ZnO and Co-doped ZnO nanophotocatalysts deposited on ceramic

**Fig. 1:**
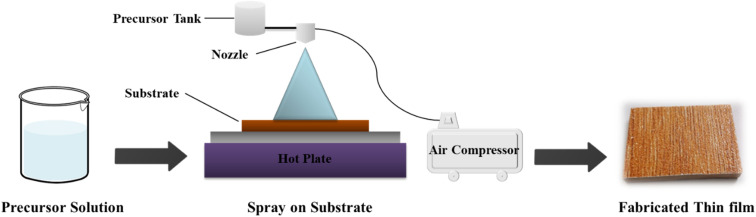
Schematic diagram of the fabrication of pristine ZnO and Co/ZnO.

In this study, the spray pyrolysis method was used to fabricate pristine ZnO and Co-doped ZnO nanoparticles on ceramic. ZnO thin films with nanostructures were deposited on ceramic substrates by spray pyrolysis of 0.1 M of zinc sulfate heptahydrate (ZnSO_4_·7H_2_O) dissolved in a solvent mixture of ethanol and distilled water. The ratio of ethanol to distilled water was retained at 2/3 in a mixture of solvents. To stop the formation of zinc hydroxide in the solution, 3–4 drops of acetic acid were added. The substrates were washed before deposition with 30% nitric acid, distilled water and finally with ethanol and then dried with the help of compressed air. The resulting solution was sprayed directly onto ceramic substrates positioned on a hot plate heated at 773 K using an infusion syringe pump at a continuous flow rate of 5 ml min^−1^. The spray solution was atomized with the help of a jet of compressed air through a nozzle positioned at 15 cm directly beyond the ceramic (substrate) (Fig. 1).^34,35^

The same procedure was used for the fabrication of Co-doped ZnO thin films only after deposition of 0.1 M of zinc sulfate heptahydrate (ZnSO_4_·7H_2_O) and 3 mM cobalt sulfate heptahydrate (CoSO_4_·7H_2_O) on a ceramic substrate. Then, the substrates were allowed to cool down to room temperature. Once they attained room temperature, the fabricated pristine ZnO and Co/ZnO nanophotocatalyst thin films were used to degrade dyes.

### 2.3. Characterization

The crystalline structures of the fabricated pristine ZnO and Co:ZnO thin films deposited on ceramic were studied by X-ray diffraction (XRD, Jeol JDX-3532, Japan). The composition, purity and elemental analysis of pristine ZnO and Co:ZnO were examined by energy-dispersive X-ray (EDX) analysis. The roughness and surface morphology of the nanophotocatalyst were determined using a scanning electron microscope (SEM). To determine the chemical composition of the material, Fourier transform infrared (FTIR) spectroscopy was carried out.

### 2.4. Computational details

Density functional theory (DFT) and the B3LYP functional (Becke's 3-parameters along with Lee–Yang–Parr correlation energy) were used along with the Gaussian 09 software package^36^ to optimize the shape of ZnO and Co/ZnO. The LanL2DZ basis sets were employed for the O^37^ and Zn^38^ atoms. To balance the charge of ZnO clusters, hydrogen (H) atoms have saturated the free bonds between the O and Zn atoms. All theoretical studies in this work used relaxed cluster atoms, with the exception of hydrogen atoms that were evaporating. In prior research, the B3LYP functional produced realistic findings for tiny ZnO clusters,^39,40^ and it has consistently provided accurate band gap predictions for a range of metal oxides. The same basis set was used for all clusters' geometry optimization and molecular orbital calculations. Additionally, frontier molecular orbitals were assessed using the optimized structural parameters. The computed energies for the lowest unoccupied molecular orbital (LUMO) and the highest occupied molecular orbital (HOMO) were used to calculate the band gaps for all clusters.^41^

Gauss View 6.0.16 was used to display the optimized structures. To guarantee that the optimized structures are at local minima, frequency calculations utilizing normal mode analysis were carried out. Theoretical calculations have mirrored experimental investigations into metal oxides. Density functional theory (DFT) and other theoretical techniques allowed for the optimization of ZnO shape,^42–44^ the analysis of band gaps, and the investigation of the spectroscopic characteristics (IR and Raman) of metallic oxides.^45^ In order to make sure that the equilibrium geometry does not have any fictitious frequencies, vibrational frequencies were calculated using the optimized structural parameters. The basis sets for geometry optimization were used to do these vibrational frequency computations.^46^

The effect of doping on optical and structural properties was studied in Co:ZnO nanoparticles. It was also investigated how Co doping impacts the HOMO and LUMO levels, band gap, and zinc oxide structure.

### 2.5. Photocatalytic degradation study

The photocatalytic activity of the fabricated 3% Co:ZnO nanophotocatalyst thin film deposited on ceramic was determined under sunlight. Methylene blue solutions of different concentrations were prepared. In the photocatalytic experiment of MB, 250 ml dye solution was taken in a beaker with dipped Co:ZnO deposited on ceramic with an appropriate amount of oxidant (H_2_O_2_). The samples were subjected to sunlight and scanned under UV-vis light. The percentage degradation of samples was determined using eqn (1):1
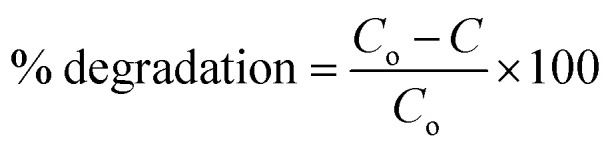


The values of water quality parameters such as COD,^47^ BOD^48^ and TOC^49^ were determined by applying the standard protocol,^50^ and the reusability of the synthesized catalyst was also examined.

### 2.6. Experimental design by the RSM method

The effect of pH, dye conc., oxidant conc. and irradiation time was investigated by applying response surface methodology (RSM). RSM is a statistical and mathematical method that aids in analyzing the importance of different operational variables, design of experiments, the investigation of parameter interactions and process optimization.^51^ The most popular method for designing environmental processes in RSM is central composite design (CCD), which offers good understanding of the interactions between various components, while requiring fewer experimental runs. Axial points, cube points and center points from factorial design are necessary for CCD. The total number of experimental runs required can be calculated as 2^*k*^ + 2*k* + *C*_o_, where *k* is the number of factors, 2^*k*^ is the number of cubic runs, 2*k* is the number of axial runs, and *C*_o_ is the number of runs at the centre point.^52^ This study showed that the central composite design comprises 6 replicated runs at the central point, 8 axial point runs, and 16 runs at the whole factorial design. In Table 1, independent variable parameters are represented as *X*_1_, *X*_2_, *X*_3_ and *X*_4_ and the levels and ranges of parameters are given. To fit the experimental findings, a quadratic model was designed.

2 *Y* = *b*_0_ + *b*_1_*X*_1_ + *b*_2_*X*_2_ + *b*_3_*X*_3_ + *b*_4_*X*_4_ + *b*_12_*X*_1_*X*_2_ + *b*_13_*X*_1_*X*_3_ + *b*_14_*X*_1_*X*_4_ + *b*_23_*X*_2_*X*_3_ + *b*_24_*X*_2_*X*_4_ + *b*_34_*X*_3_*X*_4_ + *b*_11_*X*_12_ + *b*_22_*X*_22_ + *b*_33_*X*_32_ + *b*_44_*X*_42_

where ‘*Y*’ represents the predicted response of MB by the CCD-RSM, *b*_0_ the constant coefficient, *b*_1_, *b*_2_, *b*_3_ and *b*_4_ the linear effect coefficients, *b*_12_, *b*_13_, *b*_14_, *b*_23_, *b*_24_ and *b*_34_ the interaction effect coefficients, *b*_11_, *b*_22_, *b*_33_ and *b*_44_ the quadratic effect coefficients, and *X*_1_, *X*_2_, *X*_3_, and *X*_4_ the independent variables.^53^

**Table 1:** Central composite design for different independent variable parameters

Factor	Variables	Units	Low actual	High actual
*X*_1_	pH		5	9
*X*_2_	Dye conc.	ppm	5	15
*X*_3_	Oxidant conc.	mMol	10	50
*X*_4_	Irradiation time	Hours	1	5

## 3. Results and discussion

### 3.1. Characterization

#### 3.1.1 Energy-dispersive X-ray (EDX) analysis

**Fig. 2:**
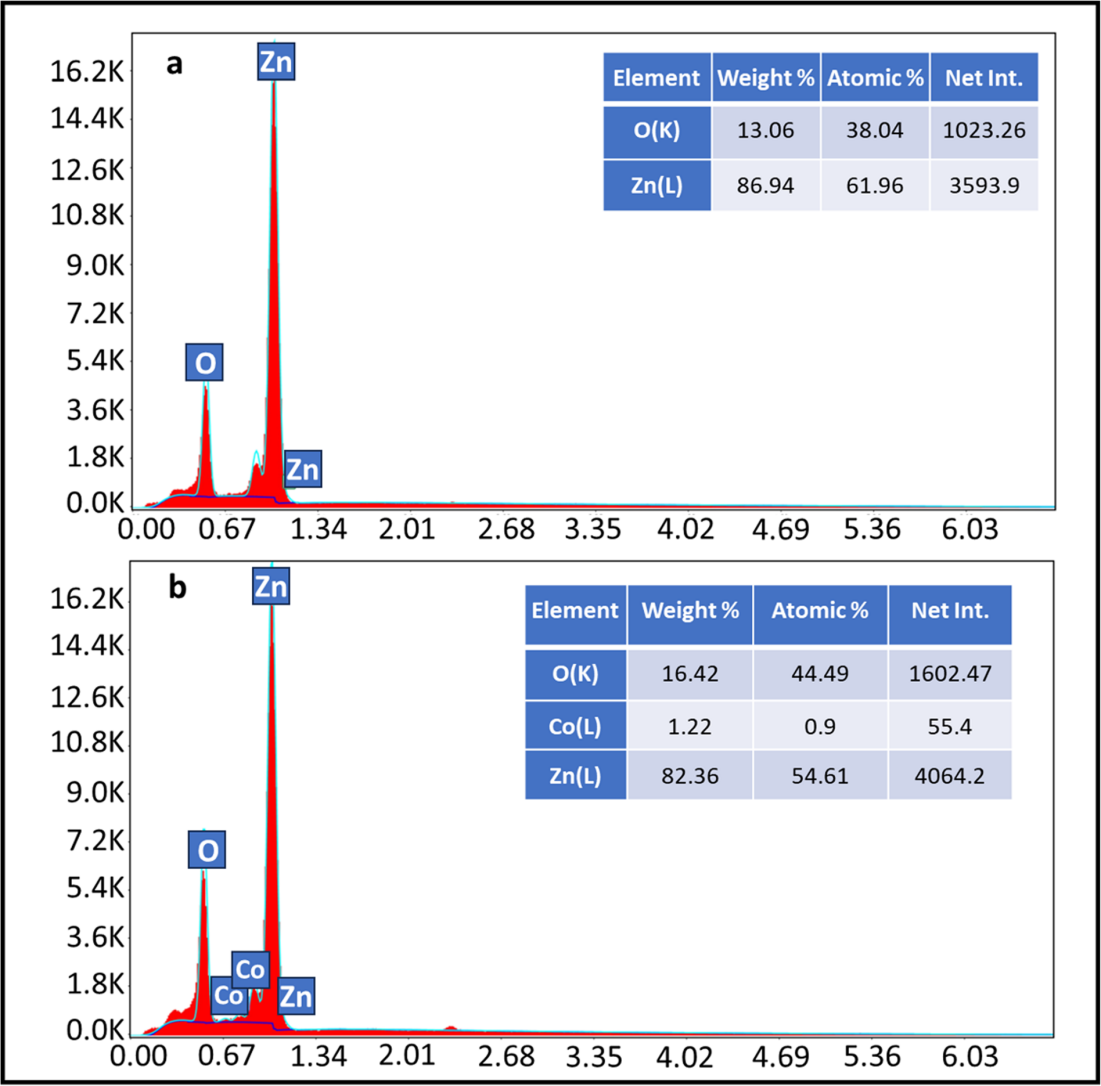
EDX analysis of (a) pristine ZnO and (b) Co-doped ZnO.

Homogeneity and elemental analysis of pristine ZnO and Co:ZnO were verified by EDX (energy-dispersive X-ray) analysis. In the EDX spectrum of pristine ZnO, the sample was reported to contain Zn and O, with two peaks of varying intensities for zinc and one peak for oxygen, as shown in Fig. 2, while cobalt, zinc, and oxygen were all found in Co-doped ZnO samples. Fig. 2 confirms the presence of Co in the ZnO matrix along with Zn and O by EDX investigation. Energy-dispersive X-ray spectroscopy (EDX) data with their corresponding percentage composition are depicted in the figure.

#### 3.1.2 Scanning electron microscopic (SEM) analysis

**Fig. 3:**
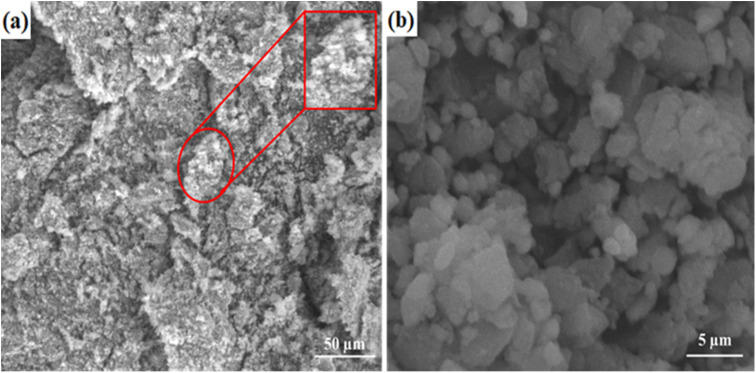
SEM analysis of (a) pristine ZnO and (b) Co-doped ZnO.

Fig. 3 represents the SEM investigation of the surface morphology of the fabricated nanophotocatalysts. The stacked thin sheet architecture of the Co-doped ZnO nanophotocatalyst is shown in Fig. 3(b). The aggregated, porous structures with a semi-round form were seen in the SEM image. As shown, bulk formations were made by piling conventionally coiled, two-dimensional sheets on top of one another.

#### 3.1.3 X-ray diffraction analysis (XRD) of fabricated Co-doped ZnO

**Fig. 4:**
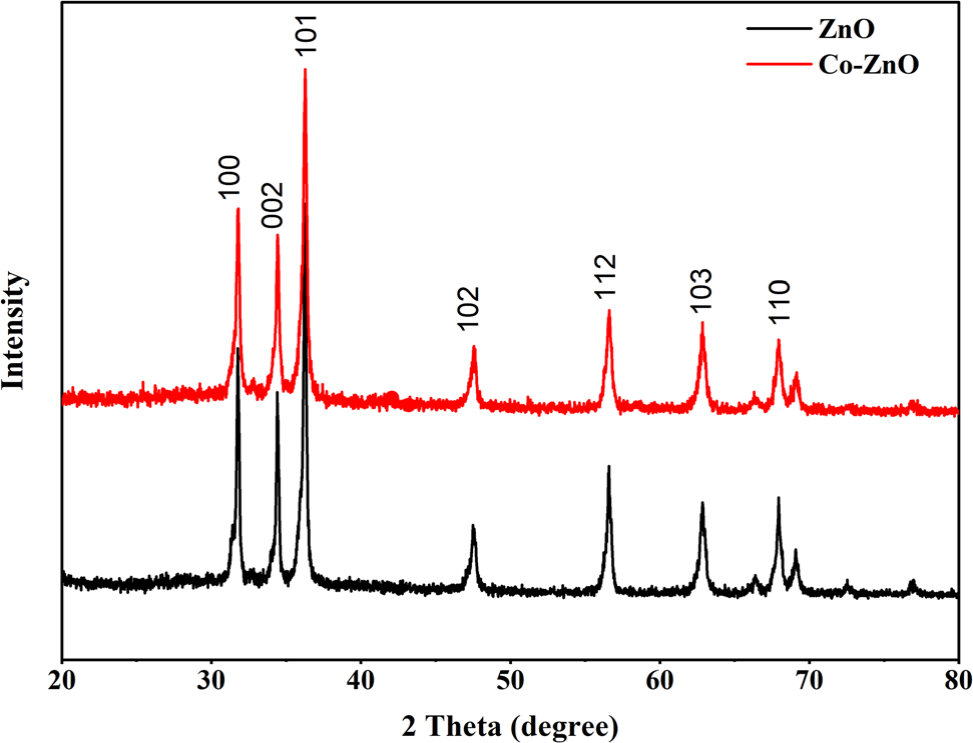
XRD spectra of pristine ZnO and Co-doped ZnO.

By using the X-ray diffraction spectroscopy technique, the purity and phase of the fabricated samples were studied. Fig. 4 shows the principal diffraction peaks for ZnO and Co:ZnO observed at 2*θ* values of 31.76°, 34.50°, 36.34°, 47.59°, 56.66°, 62.93°, 67.8°, and 68.8° with the resulting crystal planes of (100), (002), (101), (102), (112), (103) and (110). The measured rays are in perfect agreement with the typical X-ray diffraction (XRD) pattern of the hexagonal wurtzite structure of zinc oxide. A modest decrease in intensity is seen in the X-ray diffraction patterns of the Co:ZnO nanophotocatalyst, which confirms the successful metal ion doping into ZnO.^54^ The lack of impurity peaks in the patterns of the Co:ZnO nanophotocatalyst confirms that the substitution of Co ions has not changed the structure of ZnO. Due to the near proximity of the ionic radii of Co^2+^ and Zn^2+^, the transition metal-ions can easily replace the Zn^2+^ ions. Additionally, the replacement of Zn^2+^ with the transition metal-ions enhances the absorption of visible light and the corresponding PCE of the Co-doped ZnO nanophotocatalyst.^55^

#### 3.1.4 Fourier transform infrared (FTIR) analysis

**Fig. 5:**
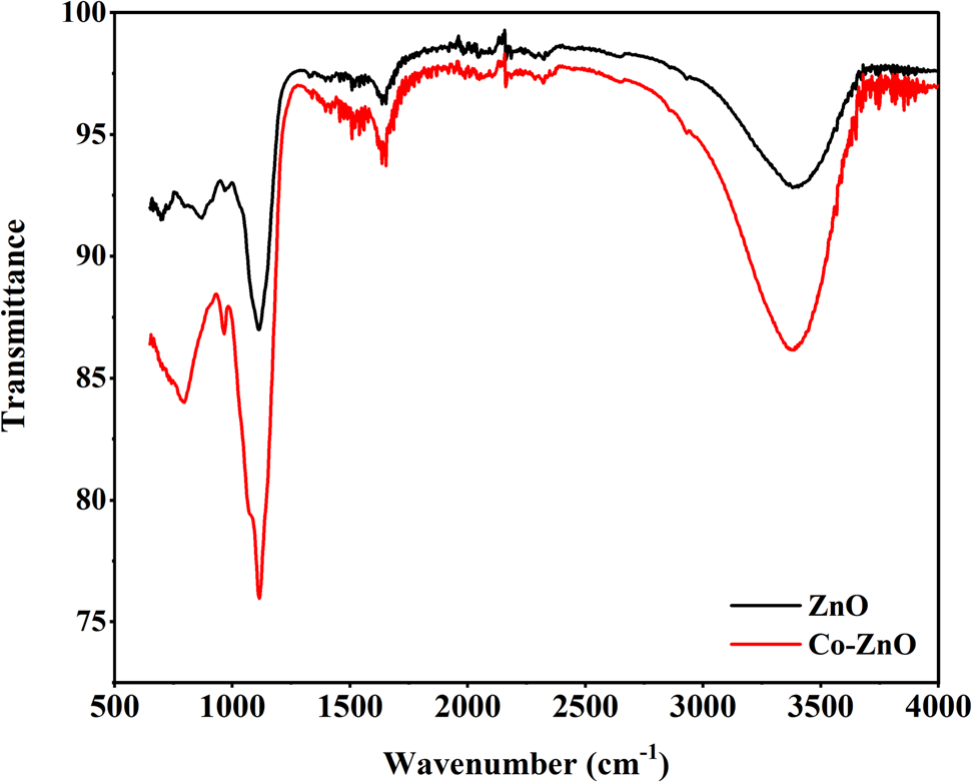
FTIR spectra of ZnO and Co-doped ZnO thin-film nanophotocatalysts.

FTIR analysis was performed for pristine ZnO and Co-doped ZnO nanoparticles in the scanning range of 700–4000 cm^−1^, and Fig. 5 depicts the results of the analysis. The peak at 3380.7 cm^−1^ is assigned to O–H stretching. Metal oxides typically display absorption bands in the fingerprint area due to inter-atomic vibrations, normally below 1000 cm^−1^. The Zn–O stretching vibration is at 700 cm^−1^. All samples contain several absorption peaks between 900 and 2900 cm^−1^. The peak at 1654 cm^−1^ corresponds to the stretching vibration of the C–O bond. An asymmetric stretching vibration of the C–O bond was at 1520 cm^−1^. Bands at 1112.6, 969.6 and 870.3 cm^−1^ correspond to the C–C stretching vibration.

#### 3.1.5 High-resolution transmission electron microscopic (HR-TEM) analysis

**Fig. 6:**
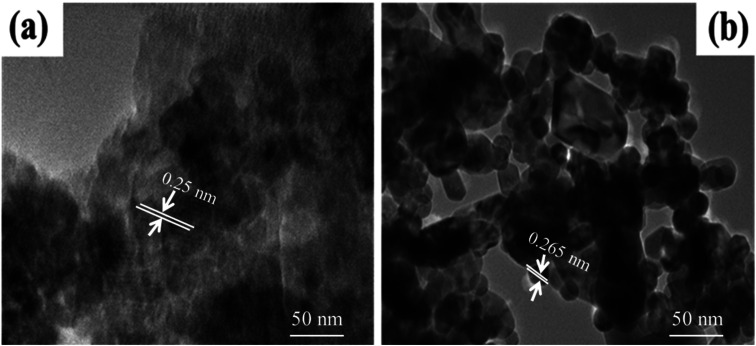
HR-TEM analysis of (a) ZnO and (b) Co doped ZnO.

A high-resolution TEM (HR-TEM) was used to determine the morphology and size of the fabricated ZnO and Co-doped ZnO nanoparticles. The TEM images of the synthesized nanoparticles are shown in Fig. 6, which depicts a clear edge around particles in the case of Co-doped ZnO nanoparticles. Analysis explains that cobalt doping changed the size and dimensions of ZnO. The pure ZnO nanophotocatalyst exhibits a large number of agglomerated particles with particle sizes ranging from 25 to 30 nm. The average particle size of the Co-doped ZnO nanophotocatalyst was reduced by 10–15 nm. The lattice fringe was calculated by measuring the distance between the dark and bright fringes (interplanar spacing) in a TEM image. The lattice spacing of ZnO was measured at 0.25 nm.

### 3.2. Computational details

**Fig. 7:**
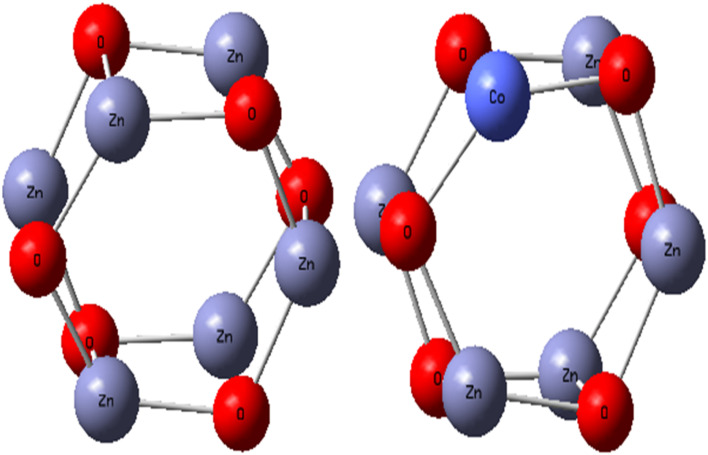
Optimized structure of pristine ZnO and Co-doped ZnO.

ZnO and Co/ZnO structures were optimized for a small cluster. The ZnO and Co-doped ZnO nanoparticles were modeled as a Zn_6_O_6_ cluster cut from the wurtzite crystal structure and then geometry-optimized, as shown in Fig. 7. Density functional theory (DFT) with B3LYP functional and LanL2DZ basis set were utilized to discuss the properties of pristine ZnO and Co/ZnO. The DFT calculations were executed with the stated lattice parameters of ZnO and the atomic locations: *a* = *b* = 3.2494 ± 0.0002 Å, *c* = 5.2054 ± 0.0002 Å, *α* = *β* = 90°, *γ* = 120°.^56^

The structural properties of Co-doped ZnO were examined and the most favourable locations of Co in the crystal structure were recognized using computational structural modelling.^57^ Because ZnO, unlike other oxides, possesses oxygen vacancies, there are three major sites for the cobalt atom in the zinc oxide structure: the substitutional point of zinc, the interstitial points of octahedral and tetrahedral voids, and one less likely position, the substitutional site of oxygen. The preferred position for cobalt was the substitutional position of zinc due to the similar ionic radii of Zn^2+^ (0.60 Å) and Co^2+^ (0.58 Å),^58^ which indicated that Co ions easily replaced Zn ions in the crystal structure. No change in the wurtzite structure of ZnO emphasizes the doping of cobalt occurs on the substitutional point of zinc.

**Fig. 8:**
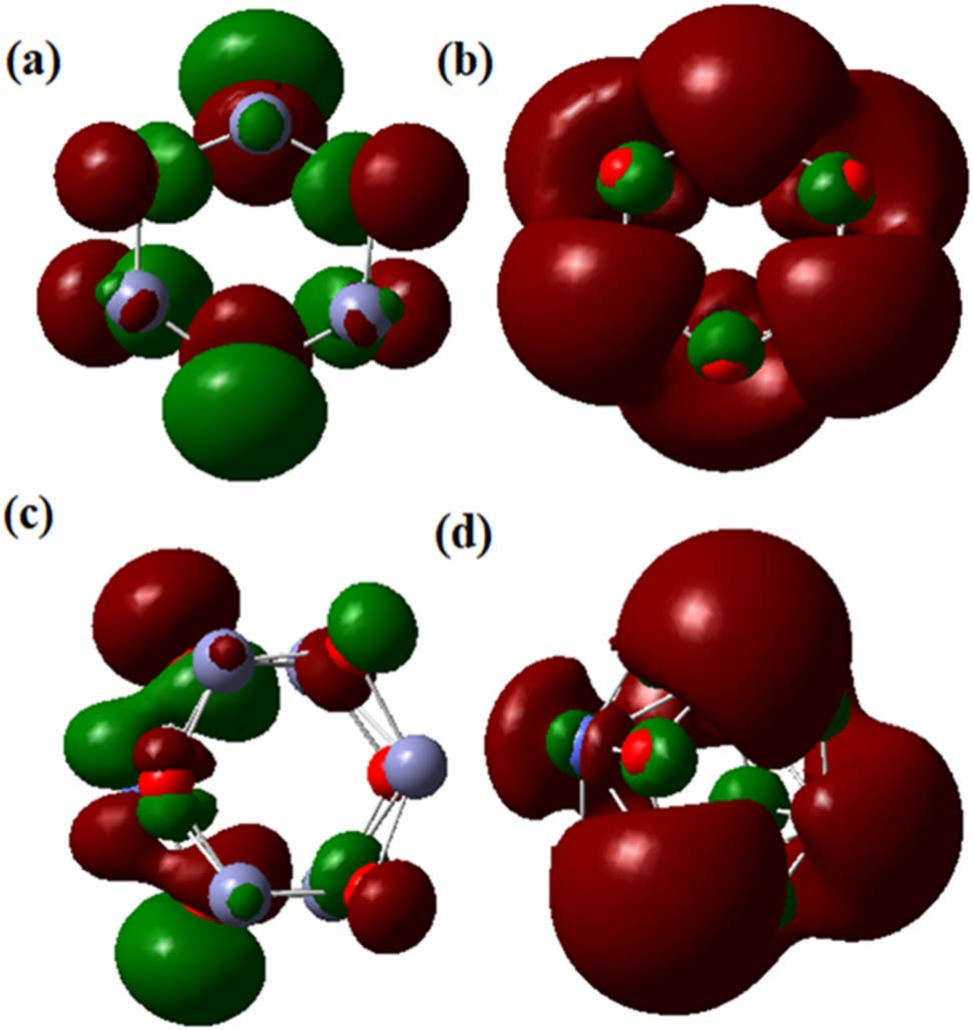
HOMO and LUMO of pristine ZnO and Co-doped ZnO nanoparticles: (a) HOMO of ZnO, (b) LUMO of ZnO, (c) HOMO of Co-doped ZnO and (d) LUMO of Co-doped ZnO.

When determining the reactivity of clusters, the highest occupied molecular orbital (HOMO) and lowest unoccupied molecular orbital (LUMO) are the vital characteristics.^59^ Using the B3LYP approach with the LanL2DZ basis set, the frontier molecular orbital forms of the Zn_6_O_6_ and Zn_5_CoO_6_ clusters were identified. According to Fig. 8(a), the electron density is primarily focused on the O atom in the Zn_6_O_6_ cluster's HOMO orbital and on the zinc and oxygen atoms in the cluster's LUMO orbital (Fig. 8(b)). In the HOMO of the Zn_5_CoO_6_ cluster, the electron density is mostly found on the zinc and oxygen atoms at the corners, while in LUMO, it is found on the outer Co atom shown in Fig. 8(c) and (d). Co-doped ZnO particles are less stable than ZnO nanoparticles and require less energy to excite an electron because they have lower band gap energies. Theoretical calculations provide results that are closely related to experimental studies. The B3LYP/LanL2DZ theoretical levels were used to quantify the Mulliken charge values. Co-doped ZnO clusters include oxygen atoms with a negative charge, suggesting that these atoms are donors. Zn and Co atoms in Co-doped ZnO nanoparticles are acceptor atoms because of their positive charges.

**Fig. 9:**
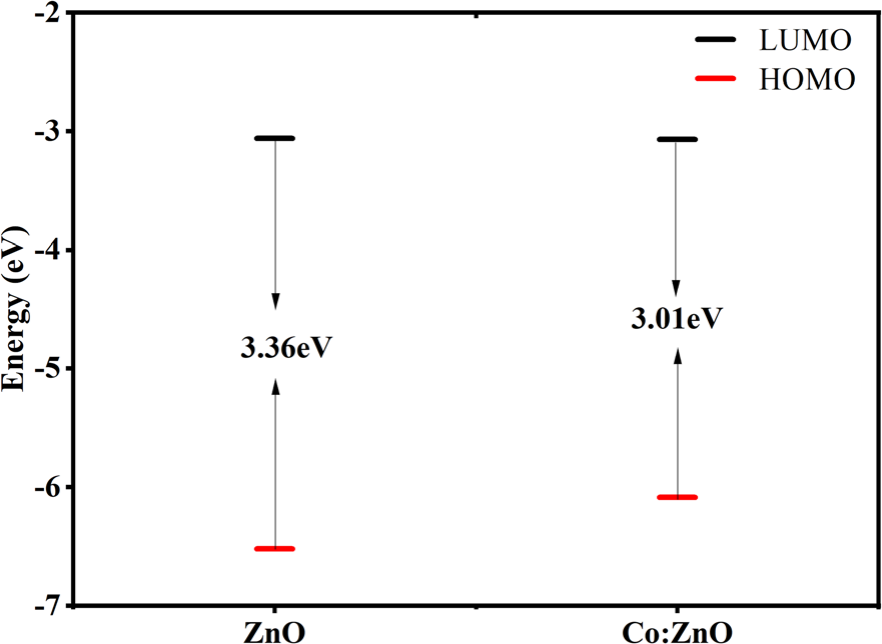
Band gap calculations of pristine ZnO and Co-doped ZnO.

The band gap, which is vital for structural stability,^60^ was measured by the energy difference between HOMO and LUMO orbits. Experimental calculations using the UV-vis spectrum yielded energy gaps of 3.34 and 2.98 eV for pure ZnO and Co/ZnO. The calculated values are in a great comparison with the experimental values as 3.36 and 3.01 eV for pure and Co/ZnO, as depicted in Fig. 9. These results clearly indicate that reduction in the band gap of doped ZnO favourable for visible ray absorption, is only 43% in case of pure ZnO. The photocatalytic mechanism is presented based on the density functional theory, with increased photocatalytic activity.

**Fig. 10:**
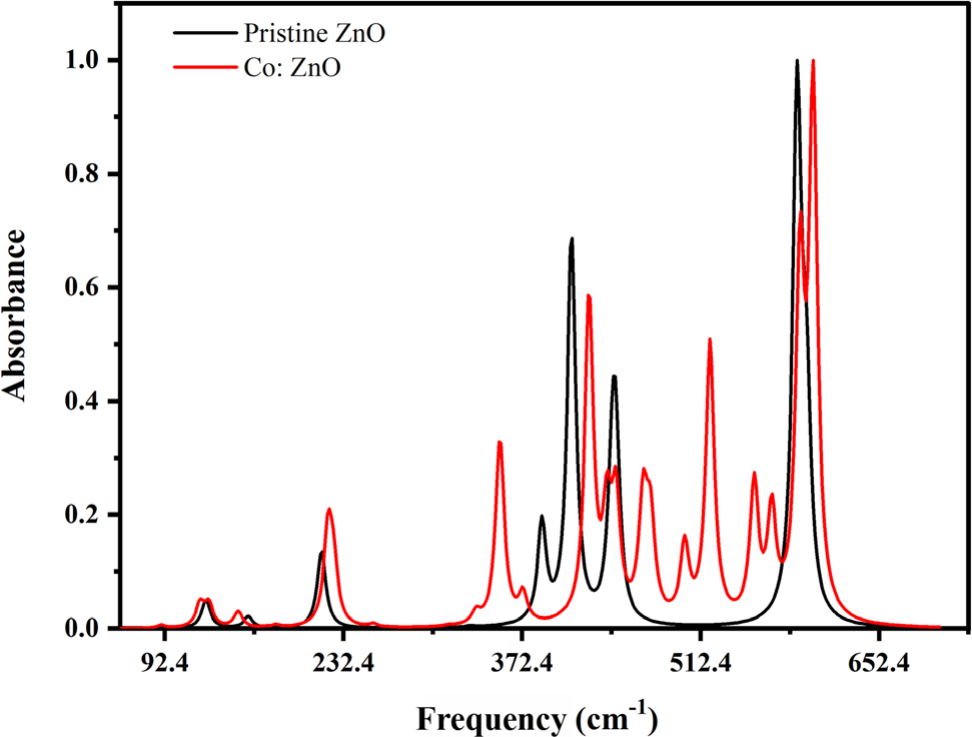
IR spectra of pristine and Co-doped ZnO nanophotocatalysts using B3LYP/LanL2DZ.

By measuring the fraction of incident IR radiation absorbed by ZnO and Co/ZnO at a specific wavelength, infrared (IR) spectroscopy gives information about molecular structures in a fabricated material. Most of the peaks of their mid-IR spectrum are above 400 cm^−1^. The ‘chemical fingerprint’ of the synthesized nanophotocatalyst shows a distinctive series of peaks with precise locations and relative intensities. The ZnO nanoparticles exhibit three bands in the IR spectrum at 400, 450 and 580 cm^−1^, which represent the features of Zn–O–Zn single-bond vibrations (Fig. 10).

Co-doped ZnO has the same three peaks at 400, 450 and 580 cm^−1^, which matched the values reported in the literature,^61^ like pure ZnO for Zn–O–Zn bonds. In addition to these bands, Co-doped ZnO nanoparticles have two peaks at 480 and 520 cm^−1^ for Co–O single bonds.

**Fig. 11:**
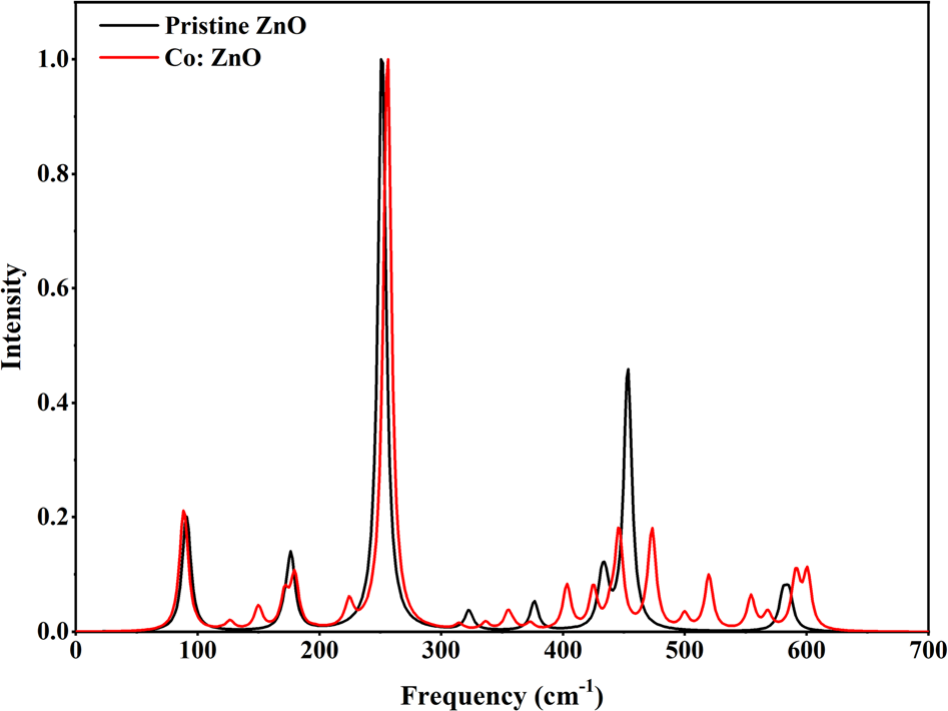
Raman spectra of pristine and Co-doped ZnO nanophotocatalysts using B3LYP/LanL2DZ.

The DFT method was used to identify the molecular structure of ZnO and Co/ZnO. Theoretical calculations were done to figure out vibrational spectra compounds and optical molecular structure. Fig. 11 shows the Raman spectra of pristine and Co/ZnO; the bands at 98, 175, 255, 440, 460 and 570 cm^−1^ correspond to various ZnO vibrational modes.^62^ The wurtzite ZnO structure was revealed by low and high scattering modes at 175 and 460 cm^−1^, respectively. These prominent peaks of ZnO are thought to be caused by oxygen atom motion in the wurtzite lattice, and a second vibrational mode at 460 cm^−1^ represented defects in the structure of the nanoparticles. This peak in Co-doped ZnO splits into two smaller peaks. The associated intensities in the doped and pure ZnO decrease with the increase in Co dopants, demonstrating that cobalt doping is entirely on zinc sites and that Co doping reduces structural flaws.

### 3.3. Statistical analysis and model development

The central composite design (CCD) of the response surface methodology was used to optimize the degradation of MB with four dependent variable parameters, namely, pH, oxidant concentration, dye concentration and irradiation time. The CCD identified potential interactions and higher order effects, and determined the suitable operating parameters. With the help of CCD, an experimental setup of 30 runs was designed and replicates of runs were used to avoid experimental errors.

Analysis of variance (ANOVA) gives accurate and efficient results when applied to experimental data. A quadratic equation of the relationship between the investigated variable parameters and the response (% degradation) was obtained.

3 % deg. = +89.20 − 3.96*A* − 1.30*B* + 2.70*C* + 25.05*D* − 1.06*AB* − 0.56*AC* − 5.31*AD* − 1.56*BC* − 1.31*BD* + 0.94*CD* − 9.03*A*^2^ − 9.50*B*^2^ − 8.34*C*^2^ − 13.44*D*^2^

The results of the experiments (ANOVA) for independent, interactive and squared effects are summarized in Table 2. According to the results, the *F*-value was 65.37 and the *p* value was <0.0001, which suggests that the model is significant. The lack of fit of the model was insignificant for MB as *p* value = 0.0657 and *F*-value = 4.12, which strongly indicates that the given model is perfectly aligned with the experimental data, and the response is significantly affected by the independent variables. Non-significant lack of fit is good.

**Table 2:** ANOVA for response surface quadratic model

Sources	Sum of squares	Df	Mean square	*F*-Value	*p*-Value prob > *F*	
Model	19 649.31	14	1403.52	65.37	<0.0001	Significant
*A*-pH	376.04	1	376.04	17.51	0.0008	
*B*-Dye conc.	39.06	1	39.06	1.82	0.1974	
*C*-Oxidant conc.	155.17	1	155.17	7.23	0.0168	
*D*-Irradiation time	12 494.67	1	12 494.67	581.95	<0.0001	
*AB*	18.06	1	18.06	0.84	0.3735	
*AC*	5.06	1	5.06	0.24	0.6343	
*AD*	451.56	1	451.56	21.03	0.0004	
*BC*	39.06	1	39.06	1.82	0.1974	
*BD*	27.56	1	27.56	1.28	0.275	
*CD*	14.06	1	14.06	0.65	0.431	
*A* ^2^	2301.03	1	2301.03	107.17	<0.0001	
*B* ^2^	2163.04	1	2163.04	100.75	<0.0001	
*C* ^2^	1360.61	1	1360.61	63.37	<0.0001	
*D* ^2^	3154.23	1	3154.23	146.91	<0.0001	
Residual	322.06	15	21.47			
Lack of fit	287.22	10	28.72	4.12	0.0657	Not significant
Pure error	34.83	5	6.97			
Cor total	19 971.37	29				
*R*-Squared	0.9839					
Adj *R*-squared	0.9688					
Pred *R*-squared	0.9322					
Adeq precision	22.277					

The correlation coefficient (*R*^2^) value ranges from 0 to 1 and can be used to assess how well the suggested model fits the experimental results. When *R*^2^ is closer to 1, the regression equation will more closely match the experimental data. The percentage of MB degradation, given by the regression equation, is often estimated using the value of *R*^2^. With an *R*^2^ value of 0.9839, the suggested regression equation could be useful in predicting the percentage of MB deterioration that will occur within the experimental range. In this investigation, the adjusted *R*^2^ value of 0.9688 was reasonably near the *R*^2^ value of 0.9839, as shown in Table 2. Additionally, the adjusted *R*^2^ value of 0.9688 was in good agreement with the predicted *R*^2^ value of 0.9322. A ratio higher than 4 is considered acceptable when estimating the signal-to-noise ratio, and in our investigation, adequate precision was 22.277, which denotes a favourable signal for the quadratic model. The recommended model can therefore be used to navigate the design space.

**Fig. 12:**
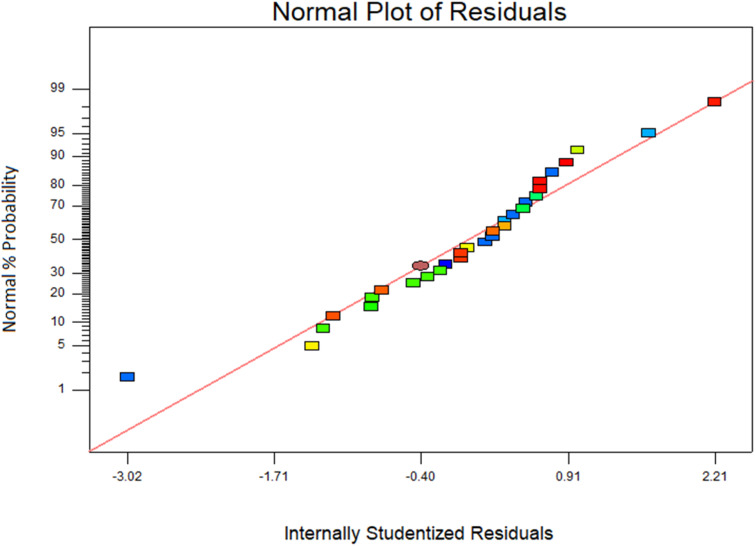
Normal plot of residuals.

Additionally, a coefficient of variation (CV) of 7.8%, which was less than 10%, might be used to illustrate the excellent reliability and precision of experimental work. The accuracy of the model was checked graphically by plotting % probability against internally studentized residuals. The normal probability plot was used to test whether the data were distributed normally or not. Data were normally distributed if all the residual points were approximately on a straight line. If residual points were not on a straight line and scattered on the plot, which indicates a departure from normality. Fig. 12 represents that all the data points form a straight line, which depicts the normality of the residual points.

### 3.4. Response surface modelling of MB degradation

**Fig. 13:**
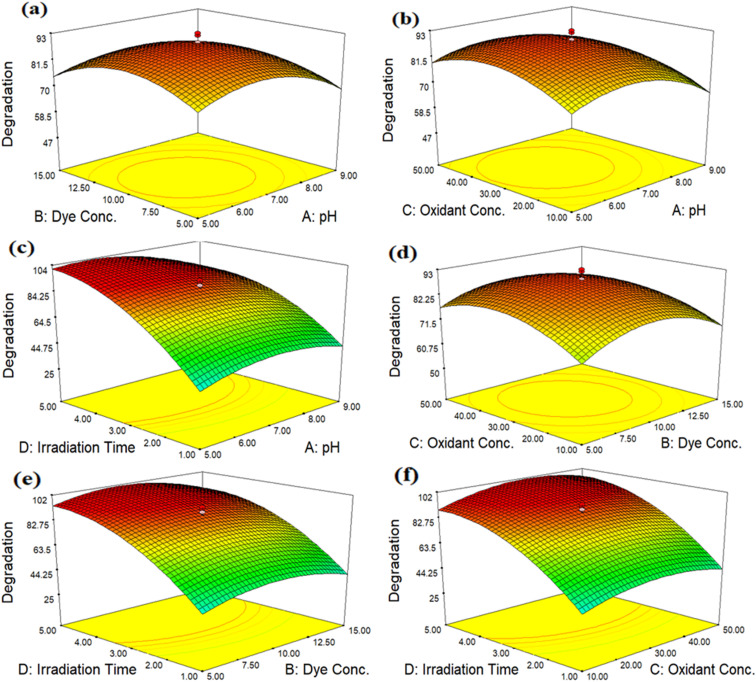
Three-dimensional plot of dye degradation using Co-doped ZnO, (a) (dye conc./pH), (b) (oxidant conc./pH), (c) (irradiation/pH), (d) (oxidant conc./dye conc.), (e) (irradiation time/dye conc.) and (f) (irradiation time/oxidant conc.).

The interaction between four independent variables, namely, pH, dye concentration, oxidant concentration and irradiation time, is revealed in Fig. 13(a)–(f). The 3D response surface plots are commonly used to establish an understanding of the types of interactions between independent parameters, which are used to increase the efficiency of the treatment. The 3D response surface plots in the form of graphical illustrations are used to find the optimum conditions of independent parameters. Two components were kept constant in each 3D plot, while the other two were allowed to change in a certain range.

Fig. 13(a) demonstrates the effect of pH and dye concentration on MB degradation. The pH level has a substantial impact on how quickly MB degrades. Degradation of MB increased when the pH value changed from acidic to neutral (5–7) and then decreased when the pH of the sample was increased (7–9). Degradation increased as the dye concentration increased from 5 to 10 ppm, but after that point, further increases in dye concentration did not affect the rate of degradation. Fig. 13(b) represents the effect of pH and oxidant concentration. The concentration of oxidant is a significant factor that has great impacts on MB degradation in a practical way. In addition to accelerating the production of HO˙ radicals, the addition of H_2_O_2_ also prevents the recombination of e^−^/h^+^ pairs. Degradation increased as the oxidant concentration increased from 10 to 30 mM, but the rate of degradation was unaffected by further increases in oxidant concentration after that. Fig. 13(c) illustrates the effect of pH and time. The irradiation time is a different element that also reasonably affects MB deterioration. Degradation increased from 1 to 3 hours of irradiation; however, the degradation rate did not change as the period continued. Fig. 13(d) reveals the effect of dye and oxidant concentrations. Due to its high oxidation potential (1.3 eV), H_2_O_2_ facilitates the oxidation process carried out by the photocatalyst. The Co-doped ZnO nanophotocatalyst and direct sunshine radiations triggered the *in situ* production of HO˙ radicals, which oxidized MB. In order to accelerate degradation and prevent electron/hole (e^−^/h^+^) recombination, HO˙, a more potent oxidizing agent, increased the secondary oxidation reaction. The degradation of the 10 ppm solution was accelerated when the oxidant concentration increased from 10 to 30 mM. The highest degradation is observed at 10 ppm of MB at 30 mM H_2_O_2_. At higher levels of H_2_O_2_, the % degradation of MB decreased. The computed results indicate that as the concentration rises, the % degradation falls. Fig. 13(e) shows the effect of dye concentration and irradiation time. The concentration of dye plays a significant role in degradation. It has been shown that degradation increased at lower concentrations of dye, as the irradiation time increased from 1 to 3 hours. After that, no significant increase was noted. At higher concentrations of dye, the rate of degradation decreased. It was observed that when the MB concentration increased, more MB molecules were loaded onto the surface of the nanophotocatalyst and more MB molecules also retrieved OH˙ radicals from the same locations. In turn, this reduced the production of OH˙ radicals and O^2−^, which minimized dye degradation at higher concentrations.

The effect of irradiation time and oxidant concentration is described in Fig. 13(f). MB deterioration is also reasonably impacted by the irradiation time and oxidant concentration. Degradation accelerated from 1 to 3 hours of irradiation onward, although the degradation rate did not change as the exposure time continued to extend. The best conditions for the degradation of MB are 30 mM oxidant and a 3 hours radiation period.

**Fig. 14:**
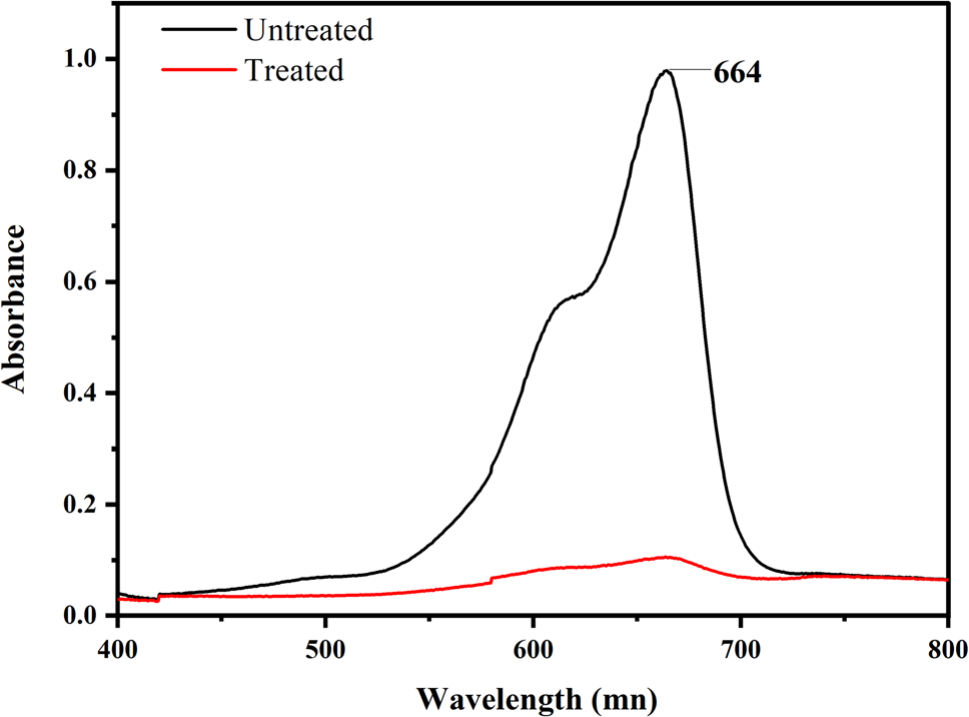
Absorption spectra of the degradation of MB.

The primary goal of the optimization method was to identify a set of variable levels that would cause the Co-doped ZnO nanophotocatalyst to degrade MB dye with the most significant amount of efficiency. In the range of investigated variables, the optimal operating conditions were attained to reach the precise point that maximizes the efficiency. From the 3D plots, the optimum values for dependent variables (pH, dye conc., oxidant conc. and irradiation time) were obtained, as pH 7, 10 ppm dye conc., 30 mM oxidant conc. and 3 hours of irradiation time. According to the data, the most significant amount of MB deterioration under ideal conditions was 93%. The degradation efficiency of the fabricated Co-doped ZnO nanophotocatalyst was compared with previous studies in Table 3. Fig. 14 demonstrates the UV/vis spectra of MB degradation under optimum conditions.

**Table 3:** Comparison of % degradation reported in the current study with that reported in previous studies

S. no.	Catalyst	Dye	Source of light	Degradation%	Exposure time	Ref.
1	Co:ZnO thin film	Crystal violet	Under sunlight	85	210 min	63
2	Co:ZnO nanoparticles	Methylene blue	Under visible light	65	300 min	35
3	Co:ZnO nanowires	Methyl orange	Under visible light	71	300 min	64
4	Co:ZnO nanowires	Methyl orange	Under 50 W Xe light	59	75 min	65
5	Co:ZnO nanorods	Rhodamine-B	Under artificial solar light	66.5	100 min	66
6	Co:ZnO film	Rhodamine-B	Under visible light	97	120 min	28
7	Co:ZnO thin film	Methylene blue	Under sunlight	93	180 min	Current work

**Fig. 15:**
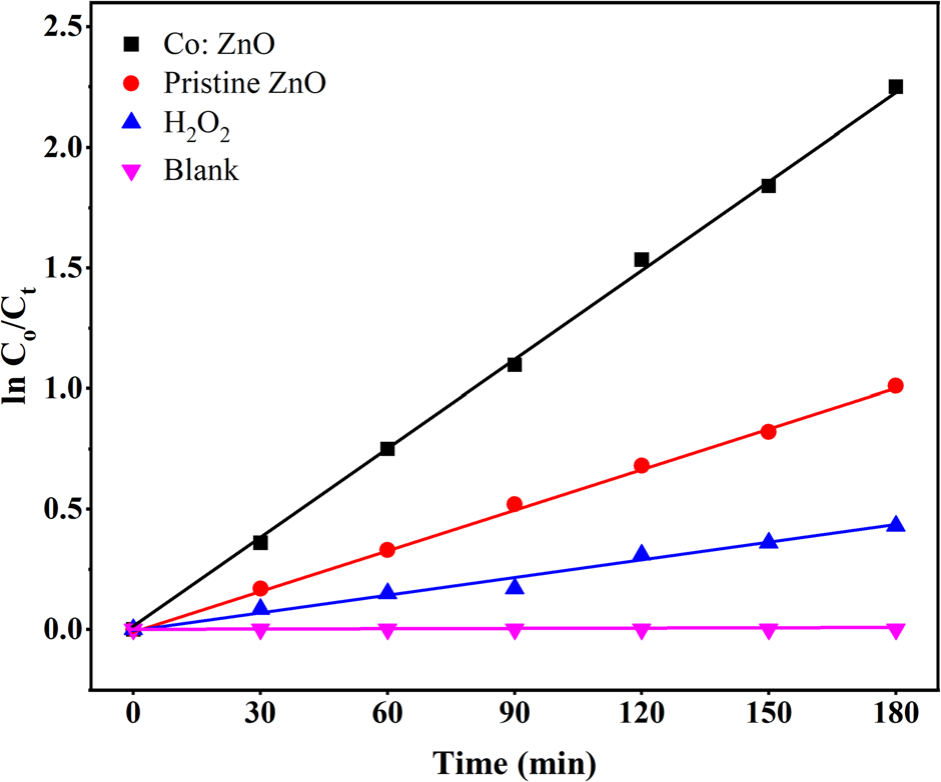
Kinetic model of the degradation of MB.

A pseudo-first-order reaction kinetic study was performed to evaluate the photocatalytic efficiency of the pure ZnO thin film and Co:ZnO thin film; the results are displayed in Fig. 15. The rates of the reaction were determined using the following equation:4
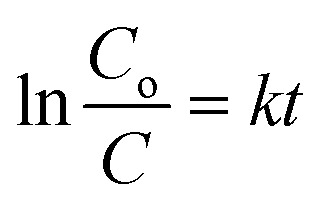
where *C*_o_ is the initial condition, *C* is the absorbance value after treatment, *t* is the time for reaction and *k* is the rate constant. The highest value of rate constant for the Co:ZnO thin film was 0.0125 min^−1^, which demonstrated that this photocatalyst exhibits better degradation of MB than pristine ZnO photocatalyst. The rate constants for pristine ZnO and a solution containing oxidants were 0.0065 min^−1^ and 0.003 min^−1^. The zero rate constant depicted that MB does not degrade in the case of the blank sample which has only MB.

The bandgap of pure ZnO is 3.34 eV and reduced to 2.98 eV when Co doping occurs, creating intermediate energy levels below the CB. These energy levels cause delay in the recombination of charge carriers, which helps to degrade MB. This demonstrates that the photocatalytic efficiency of the Co-doped ZnO nanophotocatalyst thin film is enhanced due to the involvement of large trapping sites and the subsequent separation of e^−^/h^+^ pairs. The mechanism involved in this process is as follows: when sunlight is incident on ZnO, electrons of the VB become excited and move to the CB of ZnO by creating a hole at VB (eqn (5)). By entrapping electrons from the CB of ZnO, Co^3+^ becomes Co^2+^ (eqn (6)) which forms superoxide radicals O_2_^−^ upon contact with oxygen molecules (eqn (7)). These holes and superoxide radicals react with water and generate H_2_O_2_, which is then converted into hydroxyl radicals (OH˙) (eqn (8)–(10)). These hydroxyl radicals quickly react with the dye molecules that are adsorbed on the photocatalyst surface and degrade the organic pollutants (eqn (11)).

5 ZnO + *hν* → e^−^ + h^+^

6 Co^3+^ + e^−^ → Co^2+^

7 Co^2+^ + O_2_ → O_2_^−^

8 O_2_^−^ + H_2_O → H_2_O_2_

9 H_2_O_2_ → OH˙ + OH˙

10 h^+^ + H_2_O → OH˙

11 OH˙ + dye → degradation products

**Fig. 16:**
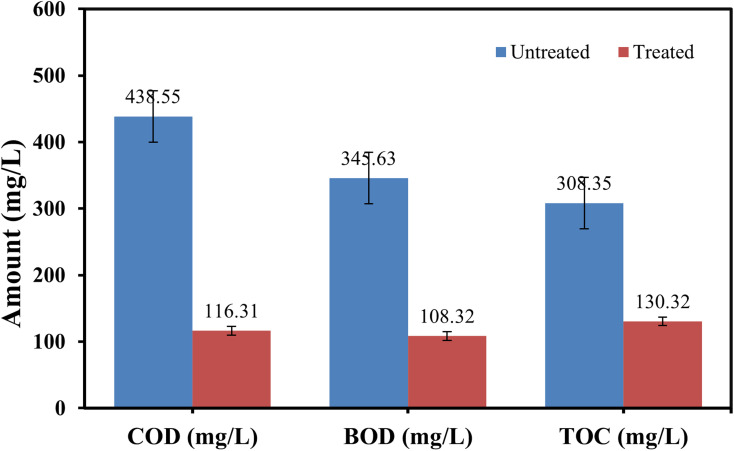
Water quality parameters before and after the photocatalytic activity.

Fig. 16 illustrates the values of water quality parameters (COD, BOD and TOC) before and after the photocatalytic activity.

The percent reduction in chemical oxygen demand, biological oxygen demand and total organic carbon was 73.5%, 68.7%, and 57.7%, respectively, which occurred after the photocatalytic degradation of MB.

**Fig. 17:**
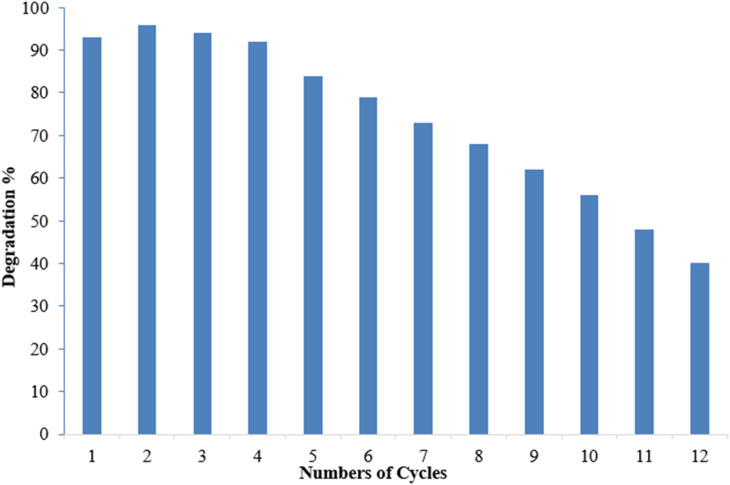
Reusability of the deposited thin film of Co-doped ZnO.

**Fig. 18:**
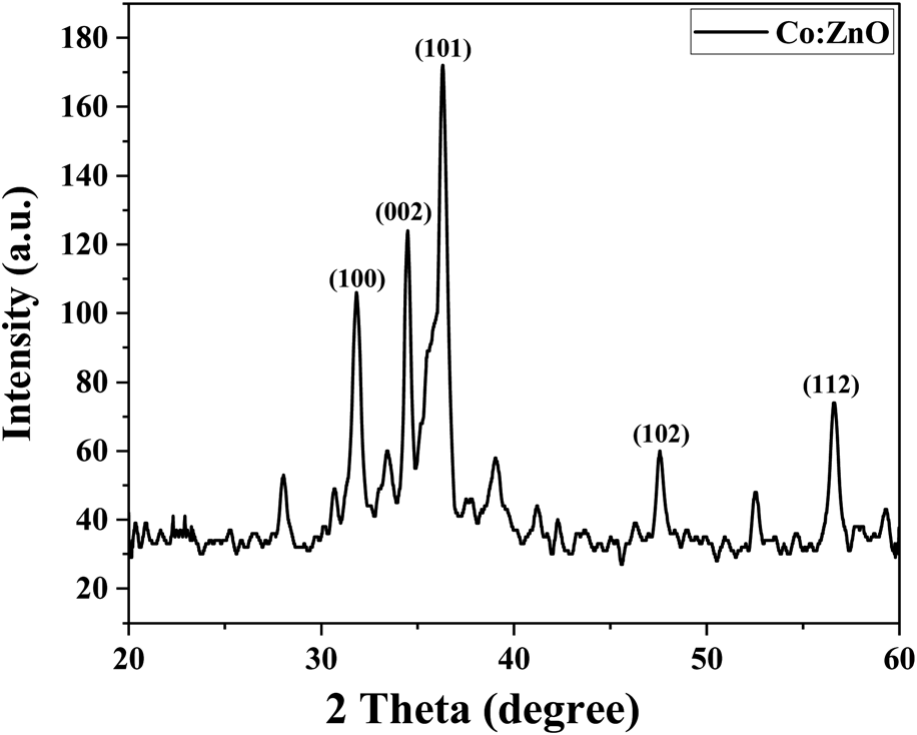
XRD pattern of Co:ZnO after reusability.

Reusability is a desirable quality of heterogeneous catalysts because it can simplify post-reaction processing and minimize the effective cost of fabricating the targeted chemical. The reusability of Co-doped ZnO nanophotocatalysts was tested under optimized conditions obtained from the RSM. pH 7, 10 ppm dye conc., 30 mM oxidant conc. and 3 hours irradiation period were the optimum conditions for MB degradation. The functionalized substrate was recycled 12 times in the current study to degrade MB. The results indicated that when the number of cycles increased, efficiency was initially retained for up to 4 cycles before steadily declining. However, even after 12 cycles, the substrate could still degrade MB by more than 40%, as shown in Fig. 17. A steady decrease in the photocatalytic activity can be due to various factors. Methylene blue residuals remained attached to ceramic areas even after rinsing and this accumulation of MB residuals might become a barrier to perform better photocatalytic activity. The stability of the photocatalyst may also be affected due to the detachment of weakly attached Co-doped ZnO nanoparticles during the rinsing process after every experiment.^67^ The XRD analysis of the fabricated Co–ZnO thin film after employment as the photocatalyst for multiple cycles was also performed to evaluate the stability and durability of the fabricated thin film (Fig. 18). The result indicated the excellent stability of the fabricated Co–ZnO thin film that makes it reusable on a commercial scale.

## 4. Conclusion

In summary, pristine and Co-doped ZnO nanophotocatalysts have been successfully fabricated by spray pyrolysis. The fabricated Co-doped ZnO nanophotocatalyst showed improved photocatalytic efficiency in comparison to the pristine ZnO nanophotocatalyst. The lack of impurity peaks in the XRD patterns of the Co-doped ZnO nanophotocatalyst confirmed that the substitution of Co ions has not changed the crystal structure of ZnO. The SEM images revealed the stacked thin-sheet architecture, significantly increasing the active sites, surface area and photocatalytic activity. The UV/vis analysis of the nanophotocatalyst demonstrated a reduction in the band gap energy from 3.34 to 2.98 eV due to Co doping, promoting the delocalization of electron–hole pairs. Maximum degradation (93%) of dye was attained by modelling MB using response surface methodology (RSM) under optimum conditions. Reduction in COD, BOD and TOC occurred at 73.5%, 68.7%, and 57.7%, respectively, by using the fabricated nanophotocatalyst. Notably, the thin film format of the catalyst offered an advantage over the powdered form, as it could be easily separated from the treated solution and reused. The functionalized nanophotocatalyst was recycled 12 times in the current study to degrade MB. In addition, we used density functional theory (DFT) with the B3LYP/LanL2DZ basis set to perform theoretical calculations for investigating the structural and optical properties. The theoretically determined band gap is in good agreement with the experimentally determined band gap values.

## Conflicts of interest

There are no conflicts to declare.

## Supplementary Material
